# Wnt/β-Catenin Signaling Inhibits Osteogenic Differentiation in Human Periodontal Ligament Fibroblasts

**DOI:** 10.3390/biomimetics7040224

**Published:** 2022-12-03

**Authors:** Ryoya Iizumi, Michiyo Honda

**Affiliations:** Department of Applied Chemistry, School of Science and Technology, Meiji University, 1-1-1 Higashimita, Tama-ku, Kawasaki 214-8571, Kanagawa, Japan

**Keywords:** periodontal ligament, Wnt-3a, osteogenic differentiation

## Abstract

The periodontal ligament is a collagenous tissue that is important for maintaining the homeostasis of cementum and alveolar bone. In tendon cells, Wnt/β-catenin signaling has been reported to regulate the expression level of *Scleraxis* (*Scx*) and *Mohawk Homeobox* (*Mkx*) gene and maintain the tissue homeostasis, while its role in the periodontal ligament is unclear. The aim of this study was to investigate the effects of Wnt/β-catenin signaling induced by Wnt-3a stimulation on the inhibition of osteogenic differentiation of human periodontal ligament fibroblasts (HPLFs). During osteogenic differentiation of HPLFs, they formed bone nodules independently of alkaline phosphatase (ALP) activity. After stimulation of Wnt-3a, the expression of β-catenin increased, and nuclear translocation of β-catenin was observed. These data indicate that Wnt-3a activated Wnt/β-catenin signaling. Furthermore, the stimulation of Wnt-3a inhibited the bone nodule formation and suppressed the expression of osteogenic differentiation-related genes such as *Runx2*, *Osteopontin* and *Osteocalcin*, and upregulated the gene expression of *Type-I collagen* and *Periostin* (*Postn*). *Scx* may be involved in the suppression of osteogenic differentiation in HPLFs. In conclusion, Wnt/β-catenin signaling may be an important signaling pathway that inhibits the osteogenic differentiation in HPLFs by the upregulation of *Scx* gene expression and downregulation of osteogenic differentiation-related genes.

## 1. Introduction

The periodontal ligament is a tissue that plays a variety of roles, including buffering mechanical stress during chewing, providing a sensory organ, and providing an immune response to periodontal disease [1,2]. Once the periodontal ligament has been lost due to periodontal disease or an accident, it is difficult for it to repair itself [3]. In order to regain the lost periodontal tissue, the most common regeneration methods today are GBR (guided bone regeneration) and GTR (guided tissue regeneration) [4]. The use of an occlusive membrane interfacing with gingival connective tissue/epithelium and a PDL/alveolar bone tissue to promote periodontal tissue regeneration is called GTR. Another application of the concept of guided regeneration involves the restoration of deficient alveolar sites for posterior implant placement. This process has been named GBR. However, as these methods rely on the cells and tissues present at the site of the defect, a complete regeneration of the periodontal ligament has not yet been established.

The periodontal ligament also plays a dual role in maintaining the soft tissue periodontal ligament while acting as a source of osteoblasts for the surrounding bone tissue. The periodontal ligament contains a multicellular population of undifferentiated mesenchymal stem cells that can differentiate into cementoblasts, osteoblasts and fibroblasts to supply the surrounding tissues, but the mechanism of differentiation is unknown [5,6,7]. Human periodontal ligament fibroblasts (HPLFs) have high alkaline phosphatase (ALP) activity, which is required for osteogenic differentiation, and express the genes related to osteogenic differentiation such as *Runx2*, *Osteocalcin* and *Osteopontin* [8,9]. Therefore, HPLFs are osteogenic fibroblasts capable of regenerating bone tissue. Better understanding of the regulation of HPLF differentiation may lead to the establishment of novel therapeutic strategies that allow regeneration of the periodontal ligament and surrounding bone tissue.

Recently, it has been reported that tendon-specific genes, *Mohawk Homeobox* (*Mkx*) [10] and *Scleraxis* (*Scx*) [11], are expressed in the periodontal ligament. The expression of *Scx* regulates the expression of *periostin* (*Postn*) in periodontal ligament-derived cells, suggesting that these tendon-specific genes are important for the maintenance of the periodontal ligament [11,12]. Periostin is a protein expressed in mechanically stressed areas such as the periosteum, periodontal ligament and Achilles tendon [13], and overexpression of periostin induces collagen production in periodontal ligament-derived cells [12], making it a commonly considered periodontal ligament marker. Tendon-specific genes have also been reported to be regulated by Wnt/β-catenin signaling in tendon-derived cells [14]. Wnt/β-catenin signaling is involved in the fate of mesenchymal stem cell differentiation, and Wnt/β-catenin signaling is regulated by Wnt proteins [15,16]. Wnt-3a, a type of Wnt protein, is used as an activator of Wnt/β-catenin signaling. Wnt-3a binds to the plasma membrane receptor Frizzled and inhibits the function of the β-catenin-degrading complex consisting of Axin, GSK-3β, APC and Dvl. By inhibiting the function of the Frizzled complex, β-catenin accumulation in the cytoplasm is stabilized and β-catenin is transferred into the nucleus. The nuclear transfer of β-catenin activates the transcriptional activity of target genes by interacting with the T-cell factor/lymphocyte enhancer factor (TCF/LEF). It has been reported that the effect of Wnt-3a on bone differentiation in periodontal tissue-derived cells differs depending on the stage of tooth development and tissue [17,18], but the effect on HPLFs remains to be elucidated.

The aim of this study was to investigate the effect of Wnt/β-catenin signaling on the osteogenic differentiation of HPLFs. HPLFs cultured in DMEM and osteogenic medium supplemented with Wnt-3a were subjected to immunofluorescence staining, Western blotting, ALP activity staining and measurement, alizarin red S staining and real time quantitative PCR. HPLFs cultured in osteogenic medium without Wnt-3a showed osteogenic differentiation independent of ALP activity and increased the expression of osteogenic differentiation related genes. The addition of Wnt-3a to the osteogenic medium activated Wnt/β-catenin signaling and suppressed osteogenic differentiation in a signal-dependent manner, suggesting that the tendon-specific gene *Scx* is involved in the suppression of osteogenic differentiation. These results indicate that Wnt/β-catenin signaling in the periodontal ligament may be involved in the suppression of osteogenic differentiation through the expression of *Scx*.

## 2. Materials and Methods

### 2.1. Materials

Chemicals and reagents were purchased from the following manufacturers.

Dulbecco’s modified Eagle’s medium–high glucose (DMEM), trypsin-EDTA, CelLytic^TM^ cell lysis reagent, and Ascorbic acid were purchased from Sigma-Aldrich (St. Louis, MO, USA). Fetal bovine serum (FBS), penicillin, streptomycin, phosphate-buffered saline (PBS, sodium chloride, potassium chloride, disodium hydrogen phosphate, and potassium dihydrogen phosphate), dimethyl sulfoxide (DMSO), Triton X-100, bovine serum albumin (BSA), Disodium Glycerophosphate 5.5 Hydrate, Dexamethasone, Alizarin Red S, Cetyl pyridinium Chloride (CPC), ImmunoStar LD, 4% Paraformaldehyde Phosphate Buffer Solution, Sirius red, LabAssay^TM^ ALP, TRAP/ALP Stain Kit and protein assay Bradford reagent were purchased from FUJIFILM Wako Pure Chemical (Osaka, Japan). Alexa Flour^®^ 488-labeled phalloidin and Alexa Flour^®^ 594-labeled goat anti-rabbit IgG1 were purchased from Invitrogen (Carlsbad, CA, USA). Anti-β-Catenin antibody (ab16051), Goat anti-rabbit IgG (H+L) antibody, HRP conjugate (SA00001-2) and α-Tubulin antibody (11224-1-AP) were purchased from Proteintech (Rosemont, IL, USA). A sample of 4′,6-diamino-2-phenylindole (DAPI) was purchased from Dojindo (Kumamoto, Japan). Human Wnt-3a protein (#5036-WN) was purchased from R&D Systems (Minneapolis, MN, USA). RNeasy Mini kit was purchased from QIAGEN (Venlo, Nederland). TaKaRa Ex Taq was purchased from Takara bio-Inc. (Shiga, Japan). Thermanox^®^ Coverslips (13 mm dia.) were purchased from Thermo Fisher Scientific (Waltham, MA, USA). LightCycler^®^ 480 SYBR^®^ Green I Master and Transcriptor First Strand cDNA Synthesis Kit were purchased from Roche (Basilea, Swiss). Human periodontal ligament fibroblasts (HPLFs) were purchased from ScienCell research laboratories (Carlsbad, CA, USA).

### 2.2. Cell Culture

HPLFs were cultured in DMEM supplemented with 10% heat inactivated FBS, 100 U/mL penicillin, and 100 µL/mL streptomycin. HPLFs were grown in a humidified atmosphere containing 5% CO_2_ at 37 °C. In this study, all experiments were conducted using the HPLFs at passage 3–5 and cultured in 24-well plates. The osteogenic differentiation medium was DMEM with 10% heat-inactivated FBS, 100 U/mL penicillin, and 100 µL/mL streptomycin, plus three osteogenic differentiation factors: 10 nM dexamethasone, 50 mg/mL ascorbic acid, and 10 mM β-glycerophosphate. The types of media are shown in Table 1.

### 2.3. Western Blotting

HPLFs were seeded at a density of 5 × 10^4^ cells/cm^2^ and cultured in DMEM. After two days, the medium was replaced by four different media and HPLFs were cultured for 14 days. The cells were lysed in CelLytic^TM^ M and homogenized by sonication. The cell lysates were separated on SDS-PAGE and a nitrocellulose membrane followed by immunoblotting with antibodies against β-catenin (ab16051, 1:4000), α-tubulin (11224-1-AP, 1:1000). Band intensities were quantified in four independent experiments for each group and were normalized by α-tubulin.

### 2.4. Immunofluorescence Microscopy

In a 24-well plate, Thermanox^®^ Coverslips (ϕ13 mm) were placed on the bottom using sterile tweezers. HPLFs were seeded at a density of 1.25 × 10^4^ cells/cm^2^ and cultured in DMEM. After one day, the medium was replaced by four different media and HPLFs were incubated for 15 min. The cells were washed twice with PBS and fixed with 4% paraformaldehyde in PBS for 15 min at room temperature. The cells were then permeabilized with 0.1% Triton X-100 in PBS for 15 min at room temperature. After rinsing with PBS twice, the cells were blocked with 3% BSA in PBS for 1 h at room temperature and incubated with a primary antibody, β-catenin (ab16051, 1:250) which was diluted in PBS overnight at 4 °C. The cells were washed with PBS twice and then stained with Alexa Flour^®^ 488-labeled Phalloidin (1:250) for F-actin, Alexa Flour^®^ 594-labeled goat anti-mouse IgG (1:250) for β-catenin and DAPI (1:500) for the nucleus diluted in PBS for 1 h at room temperature in the dark. The cells were washed with PBS and then examined by fluorescence microscopy (BZ X-710, Keyence, Osaka, Japan).

### 2.5. Alkaline Phosphatase Staining

HPLFs were seeded at a density of 5 × 10^4^ cells/cm^2^ and cultured in DMEM. After two days, the medium was replaced by four different media and HPLFs were cultured for 14 days. The cells were stained with alkaline phosphatase (ALP) stain according to the manufacture’s protocol.

### 2.6. Alkaline Phosphatase Activity

HPLFs were seeded at a density of 5 × 10^4^ cells/cm^2^ and cultured in DMEM. After two days, the medium was replaced by four different media and HPLFs were cultured for 14 days. HPLFs were collected and stored at −80 °C. The cells were lysed in CelLytic^TM^ M and homogenized by sonication. This experiment was conducted using LabAssay^TM^ ALP and following the manufacturer’s protocol. Total protein concentrations were determined by the Bradford standard method.

### 2.7. Alizarin Red S Staining

HPLFs were seeded at a density of 5 × 10^4^ cells/cm^2^ and cultured in DMEM. After two days, the medium was replaced by four different media and HPLFs were cultured for 21 days. The cells were washed twice with PBS and fixed with 4% paraformaldehyde in PBS for 15 min at room temperature. The cells were then washed twice with dH_2_O and stained with 0.1% alizarin red S solution (pH 4.1) for 1 h at room temperature in the dark. The cells were washed four times with dH_2_O and then examined by fluorescence microscopy (BZ X-710, Keyence, Osaka, Japan). Ten percent (wt/vol) cetyl pyridinium chloride was added to the 24-well plate. The optical density values were read at 570 nm.

### 2.8. Quantitative Real-Time PCR

The expression levels of *Runx2*, *Osteocalcin*, *Osteopontin*, *Type-I collagen*, *Periostin*, *Mohawk*, and *Scleraxis* HPLFs were seeded at a density of 5 × 10^4^ cells/cm^2^ and cultured in DMEM. After two days, the medium was replaced by four different media and HPLFs were cultured for 14 days. Total RNA was extracted using the RNeasy Mini kit and complementary DNA was synthesized using the Transcriptor First Strand cDNA synthesis kit. The primer sequences used in the experiment are listed in Table 2, and quantitative-PCR analysis was conducted on a LightCycler^®^ 480 Real Time PCR System (Roche Diagnostics, IN, USA) using a LightCycler^®^ 480 SYBR^®^ Green I Master. The amounts of mRNA were calculated as relative quantities in comparison to 18S mRNA and analyzed with the 2^ΔΔ^Ct method.

### 2.9. Statistical Analysis

The data were statistically analyzed for determination of the mean and the standard deviation (SD) of the mean. The Student’s t-test was carried out with a significance level of *p* < 0.05 and *p* < 0.01.

## 3. Results

### 3.1. Activation of Wnt/β-Catenin Signaling by Wnt-3a Stimulation in HPLFs

To investigate whether Wnt-3a activates Wnt/β-catenin signaling and induces the transfer of β-catenin to the cell nucleus, cells were observed using fluorescence microscopy (BZ 710, KEYENCE) 15 min after the treatment of HPLFs with Wnt-3a.

**Figure 1:**
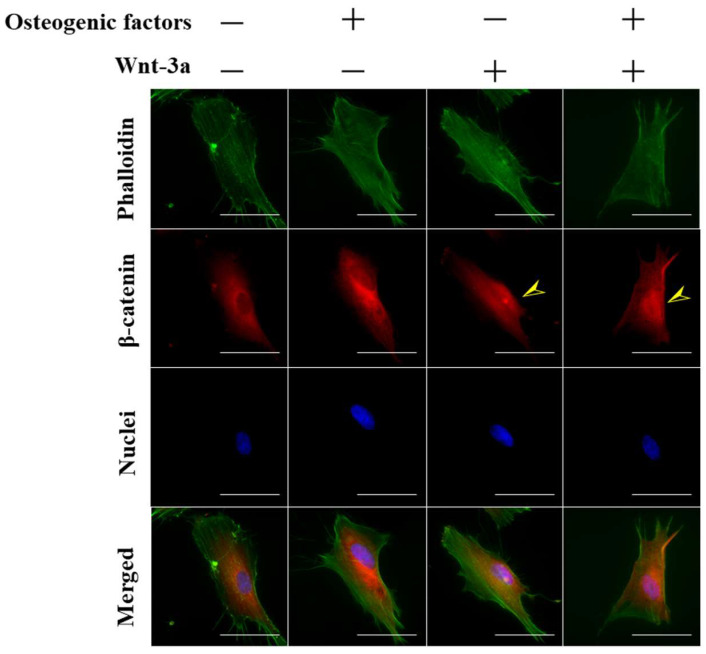
Fluorescence observation of the nuclear translocation of β-catenin upon stimulation with Wnt-3a. HPLFs were seeded at a density of 1.25 × 10^4^ cells/cm^2^ and the medium was changed after one day. Fifteen min later, cells were observed. The cells were fixed and stained with Alexa Flour^®^ 488-labeled phalloidin for actin (green), anti-β-catenin for β-catenin (red) and DAPI for cell nuclei (blue). The yellow arrows indicate the areas of nuclear migration. They were viewed through a fluorescence phase-contract microscope at 100× magnifications (scale bar: 100 μm). Nuclear translocation of β-catenin was observed in Wnt-3a-treated HPLFs, whereas that of untreated Wnt-3a was less.

When HPLFs were cultured in (−/+) and (+/+) medium supplemented with Wnt-3a for 15 min, the nuclear translocation of β-catenin was observed as indicated by yellow arrows (Figure 1). In contrast, when HPLFs were incubated in a (−/−) and (+/−) medium without Wnt-3a, β-catenin was observed to be predominantly localized in the cytoplasm rather than in the nucleus. More than 3000 HPLFs were counted after 15 min of incubation in each medium, and the results showed that (−/−): 16.2 ± 5.8%, (+/−): 11.0 ± 3.9% (−/+): 23.4 ± 7.3%, (+/+): 18.1 ± 7.0%, respectively (Figure S1). It has been reported that the nuclear-cytoplasmic transport of β-catenin is completed in a relatively short period of time (15 min) [19], and in this experiment, the nuclear translocation of β-catenin was confirmed by treatment of HPLFs with Wnt-3a for 15 min.

**Figure 2:**
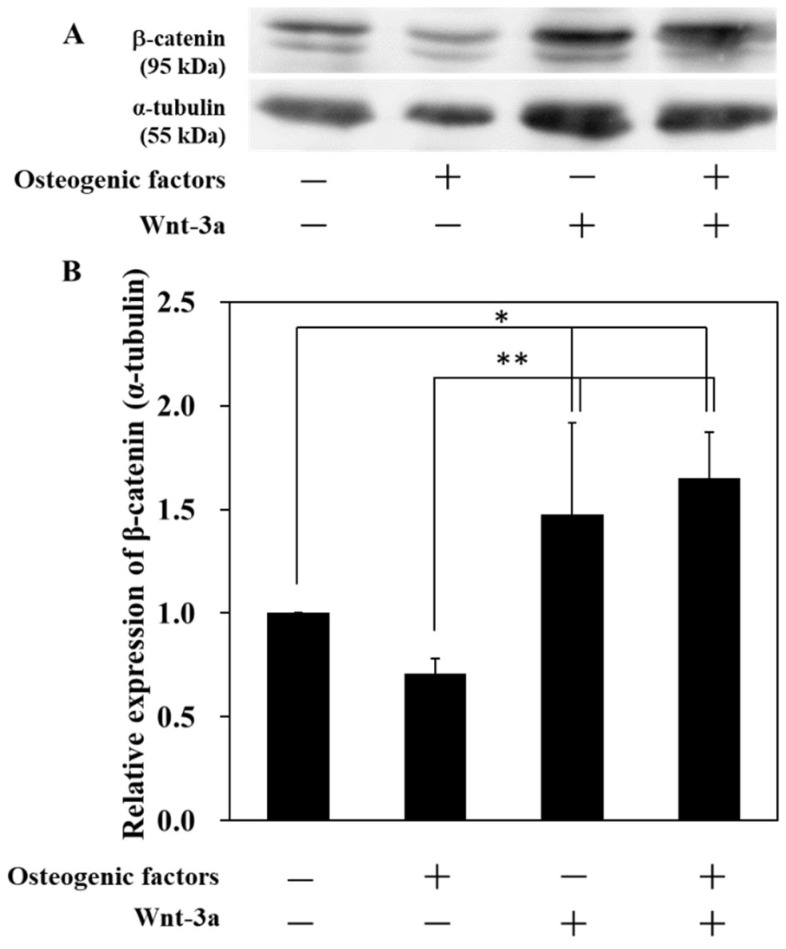
The expression levels of β-catenin after treatment with Wnt-3a. Treatment of HPLFs with Wnt-3a resulted in high expression of β-catenin and activation of Wnt/β-catenin signaling. HPLFs were treated with osteogenic medium and Wnt-3a for 14 days. (**A**,**B**) The expression of β-catenin was examined by Western blot analysis. Data represent the means ± S.D. * *p* < 0.05, ** *p* < 0.01, (n = 4).

Wnt-3a activates Wnt/β-catenin signaling by binding to the Frizzled receptor on the plasma membrane and inhibiting the Axin complex, which degrades β-catenin, thereby stabilizing β-catenin accumulation in the cytoplasm and inducing nuclear translocation of β-catenin. To further investigate whether stimulation with Wnt-3a contributes to the activation of Wnt/β-catenin signaling in HPLFs, Western blotting was performed, and the amount of β-catenin expression accumulated in the cells was measured. The expression of β-catenin in HPLFs cultured in (−/+) and (+/+) medium was significantly increased compared to those cultured in (−/−) and (+/−) medium (Figure 2). The expression of β-catenin was highest in the (+/+) medium compared to the other medium conditions.

In contrast, there was no change in β-catenin levels between (−/−) and (+/−) medium. These results indicate that HPLFs activate Wnt/β-catenin signaling by treatment with Wnt-3a. Osteogenic differentiation in periodontal ligament-derived cells is thought to involve a variety of signals, including MAPK, JNK and Erk1/2 signaling [20]. It has been reported that Wnt signaling interacts with Erk1/2 in chondrogenesis in another tissue, the mouse cranium [21]. In addition, bone marrow-derived mesenchymal stem cells from the elderly have a reduced capacity for multilineage differentiation, but an increase in β-catenin following activation of Wnt/β-catenin signaling restored their capacity for muscle differentiation [22]. Thus, Wnt/β-catenin signaling may be involved in multiple signaling and may inhibit the loss of pluripotency, as is also the case in the periodontal ligament.

### 3.2. Effect of Wnt/β-Catenin Signaling on the Production of ALP, a Marker of Early Osteogenic Differentiation

ALP staining and ALP activity were conducted on HPLFs after 14 days of culture in each medium to investigate the production of ALP, a marker associated with early bone differentiation. ALP is a hydrolytic enzyme that plays an important role in the progression of osteogenic differentiation, removing pyrophosphate, an inhibitor of the synthesis of hydroxyapatite, the main component of bone, by hydrolysis, and providing phosphate ions [23].

**Figure 3:**
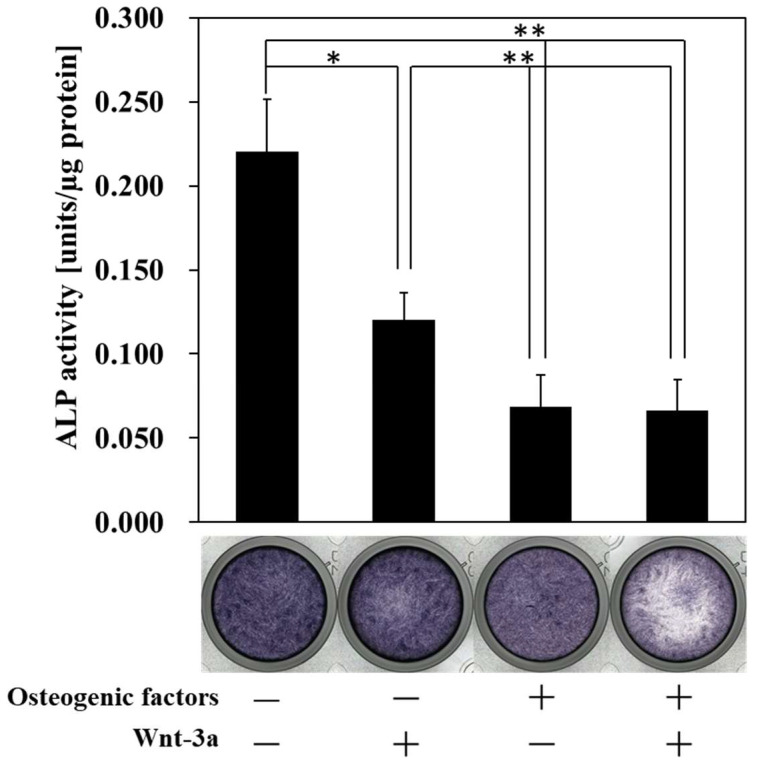
ALP activity measurement and ALP staining after treatment with Wnt-3a. HPLFs were treated with osteogenic medium and Wnt-3a for 14 days (**A**,**B**). ALP staining showed that the staining around the center was uneven when (+/+) was used. There was a significant decrease in ALP activity in (−/+), (+/−) and (+/+) compared to (−/−). And there was no significant difference between (+/−) and (+/+). ALP activity and staining were carried out using a kit. Data represent the means ± S.D. * *p* < 0.05, ** *p* < 0.01, (n = 3).

Osteoblasts are known to exhibit high ALP activity during osteogenic differentiation and high ALP activity is one of the indicators of osteogenic differentiation [24,25,26]. However, HPLFs showed high ALP activity in the absence of osteogenic differentiation, and ALP activity was suppressed as osteogenic differentiation progressed (Figure S2). ALP activity was significantly decreased in (−/+), (+/−) and (+/+) cultures compared to (−/−) cultures (Figure 3). There was no significant difference between (+/−) and (+/+). The ALP staining images were weak only in (+/+), but there was no difference in staining in the other mediums. Although ALP staining was weaker only in (+/+), there was no difference in ALP activity between (+/−) and (+/+). This suggests that the incubation of HPLFs with (+/+) increased the number of ALP-negative HPLFs, but further increased the activity of ALP-positive HPLFs, with the result that there was no change in the total amount of ALP activity. These results suggest that the ALP activity of HPLFs is suppressed by osteogenic differentiation and that Wnt/β-catenin signaling is unable to abrogate the ALP activity suppressed by osteogenic differentiation.

### 3.3. Effect of Wnt/β-Catenin Signaling on the Formation of Calcified Nodules

**Figure 4:**
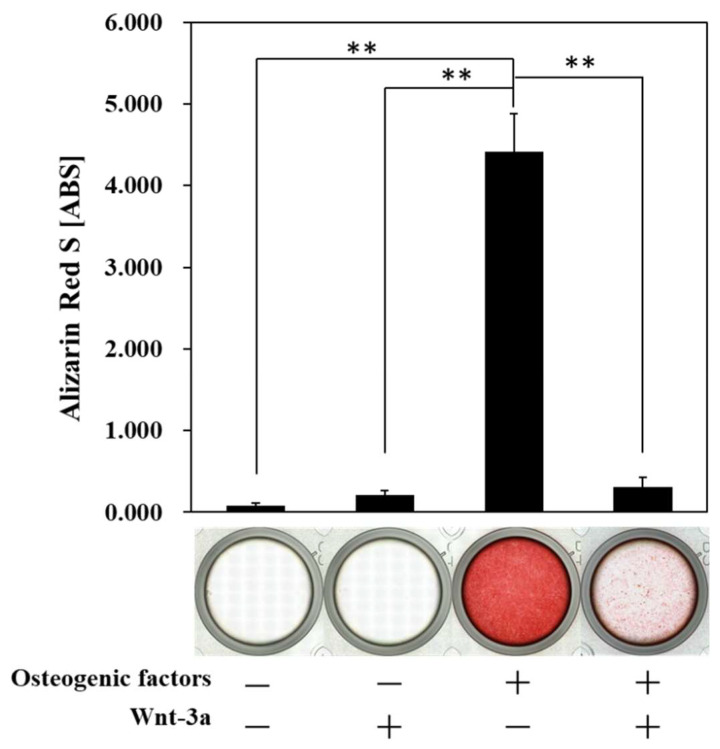
Alizarin red S staining after treatment with Wnt-3a. Cultivation of HPLFs in osteogenic medium resulted in the formation of calcified nodules in HPLFs, but Wnt-3a significantly inhibited this process. HPLFs were treated with osteoblast medium and Wnt-3a for 21 days. Data represent the means ± S.D. ** *p* < 0.01, (n = 3).

After 21 days in culture, HPLFs were stained with alizarin red S, a chelate complex with calcium. As a result, HPLFs stained red when cultured in (+/−) medium, indicating osteogenic differentiation with the formation of calcified nodules (Figure 4).

The results of ALP activity (Figure 3) indicate that ALP activity may not be involved in the osteogenic differentiation of HPLFs. Therefore, HPLFs may have the potential to differentiate into osteoblasts.

### 3.4. Effect of Wnt/β-Catenin Signaling on Gene Expression

**Figure 5:**
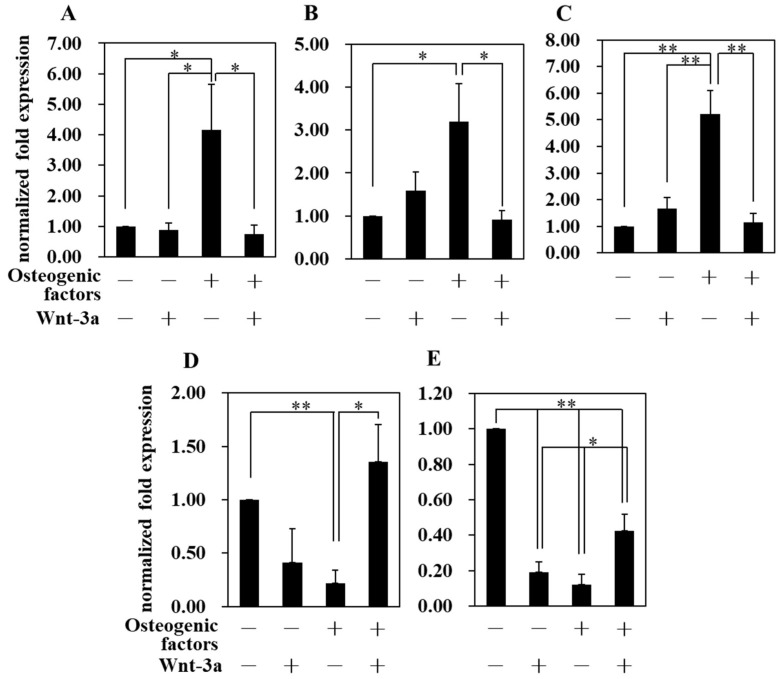
Gene expression analysis after treatment with Wnt-3a by real time quantitative PCR. HPLFs were treated with osteoblast medium and Wnt-3a for 14 days. Osteogenic differentiation-related genes ((**A**) *Runx2*, (**B**) *Osteopontin*, (**C**) *Osteocalcin*). Periodontal ligament-related genes ((**D**) *Type-I collagen*, (**E**) *Postn*). Cultivation of HPLFs in (+/−) medium increased the expression of osteogenic differentiation-related genes compared with cultivation in other mediums. (+/+) medium suppressed the upregulation of osteogenic differentiation-related genes in HPLFs cultured in (+/−) medium. The expression of periodontal ligament-related genes was, on the contrary, suppressed by (+/−) medium, whereas (+/+) medium abrogated its function. Data represent the means ± S.D. * *p* < 0.05, ** *p* < 0.01, (n = 3).

**Figure 6:**
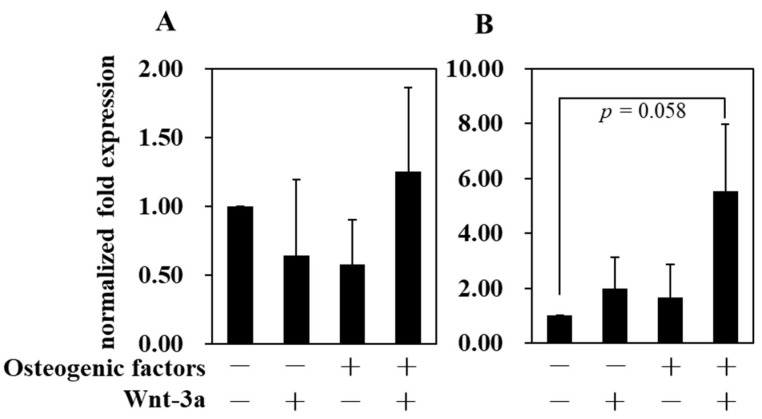
Gene expression analysis after treatment with Wnt-3a by real time quantitative PCR. HPLFs were treated with osteoblast medium and Wnt-3a for 14 days. Tendon-specific genes ((**A**) *Mkx*, (**B**) *Scx*). The expression level of the *Mkx* gene was not significantly different between the mediums, but it tended to be suppressed by (+/−) medium and increased by (+/+) medium. The expression level of the *Scx* gene showed a tendency to increase in (+/+) medium. Data represent the means ± S.D.

The expression levels of *Runx2* (Figure 5A), *Osteopontin* (Figure 5B) and *Osteocalcin* (Figure 5C) genes were significantly increased in the (+/−) medium compared to the (−/−) medium. On the other hand, the (+/+) medium significantly suppressed the expression of these osteogenic genes compared to the (+/−) medium. These results are consistent with the results of alizarin red S staining (Figure 4), which showed that activation of Wnt/β-catenin signaling induced by Wnt-3a in HPLFs suppressed osteogenic differentiation. In addition, the gene expression of the Type-I collagen was suppressed in (+/−) medium but increased in (+/+) medium. The periodontal ligament is a fibrous tissue and the Sharpey fibers present in the periodontal ligament partially penetrate the alveolar bone and cementum, thereby connecting the two tissues [27]. When osteogenic differentiation is promoted by (+/−) medium, the expression of *Type-I collagen* is suppressed (Figure 5D). This suggests that another type of collagen, but not *Type-I collagen*, may play an important role in favoring the formation of alveolar bone and cementum. When HPLFs were cultured in (+/+) medium, the expression of *Type-I collagen* was significantly increased compared to (+/+) medium and was at the same level compared to (−/−) medium, suggesting that the suppression of *Type-I collagen* expression by osteogenic induction was abrogated by Wnt-3a. Therefore, Wnt-3a was considered to be an important factor for the maintenance of periodontal ligament tissue, because Wnt-3a abolished the suppression of *Type-I collagen* expression by (+/−) medium. The gene expression level of *Periostin* (*Postn*) also displayed similar behavior to that of *Type-I collagen* (Figure 5E). Periostin (*Postn*) is a protein that is expressed at sites of mechanical stress, and the periodontal ligament is constantly exposed to the mechanical stress of mastication [28]. It has also been reported that *Postn* gene expression was increased in osteoblasts in which *Osterix* (*Osx*), a gene essential for osteoblast osteogenesis, was knocked down [29]. *Postn* is highly expressed in cells within the periodontal ligament and in osteoblasts on the surface of the alveolar bone, the site of insertion of Sharpey fibers, and is thought to be important in periodontal ligament remodeling as it is highly expressed during tooth movement [30]. Therefore, the enhanced expression of *Postn* in (+/+) medium compared to (+/−) medium suggests that *Postn* plays an important role in suppressing osteogenic differentiation and maintaining the multiple differentiation potential of the periodontal ligament. The gene expression levels of *Mkx* and *Scx*, which are tendon-specific genes, were investigated by real time quantitative PCR (Figure 6A,B). It has been reported [11] that the overexpression of *Scx* inhibits osteogenic differentiation by suppressing the expression of *Osteopontin* and *Osteocalcin* in periodontal ligament-derived cells. The relationship between Figure 5B,C and Figure 6B is similar to that reported in that case. These results suggest that *Scx* is an important gene for the suppression of osteogenic differentiation of HPLFs.

## 4. Discussion

The periodontal ligament is a soft tissue that lies between the alveolar bone and the cementum. The periodontal ligament is an important tissue for the homeostasis of periodontal tissues because it contains a wide variety of cells such as fibroblasts, osteoblasts, cementoblasts, and epithelial cells as well as mesenchymal stem cells [5,6,7]. Using the most abundant cell type, periodontal ligament fibroblasts (HPLFs), we investigated how Wnt/β-catenin signaling affects the osteogenic differentiation of HPLFs.

Immunofluorescence staining of β-catenin showed that the number of cells with nuclear transfer of β-catenin was higher in the medium supplemented with Wnt-3a (Figure 1). Although β-catenin has a molecular weight of around 90 kDa and does not contain the nuclear localization or extracellular transport signal sequences required for nuclear-cytoplasmic transport, it has been reported to be efficient in nuclear translocation [31]. It has also been reported that the C-terminal amino acid sequence of β-catenin is involved in this nuclear translocation [32] and that β-catenin nuclear-cytoplasmic transport is completed in 15 min [19]. The periodontal ligament maintains homeostasis by being Wnt-responsive [33], suggesting that HPLFs cultured in (+/+) medium maintain homeostasis in response to Wnt-3a. The expression of β-catenin in HPLFs was significantly increased in the medium supplemented with Wnt-3a compared to (−/−) and (+/−) medium (Figure 2), which was similar to the results of immunofluorescence staining (Figure 1). The increase in intracellular β-catenin and the results of fluorescence microscopy show that Wnt-3a activates Wnt/β-catenin signaling in HPLFs.

The periodontal ligament is responsible for supplying bone progenitor cells for the alveolar bone and cementum, and it has been reported that periodontal ligament-derived cells are osteogenic [9,20,34,35]. ALP activity assays (Figure 3) and alizarin red S staining (Figure 4) confirmed that HPLFs may be capable of osteogenic differentiation independent of ALP activity, which differs from what has been reported previously. ALP is a hydrolytic enzyme and is generally considered essential for bone formation, as it promotes bone tissue formation by hydrolyzing pyrophosphate, an inhibitor of hydroxyapatite formation [36,37]. However, ALP has been used as a stem cell marker to characterize iPS cells, and there are reports that ALP-negative cells cannot be induced into iPS cells [38]. It has also been reported [38] that ALP-negative cells can become ALP-positive cells by the sequential introduction of factors essential for iPS cell formation, and that stem cell markers can reach gene expression levels equivalent to those of established iPS cells. Thus, high ALP activity may function as a marker of pluripotency. In this study, we found that ALP activity of HPLFs increased in (−/−) medium in a time-dependent manner (Figure S2), and at day 14, ALP activity was significantly higher in (−/−) medium than in (+/−) medium (Figure 3). This may indicate that HPLFs have the capacity to multiply and that the induction of osteogenic differentiation results in the loss of multiplicity and a shift towards osteogenesis. Cultures of HPLFs in (+/+) medium showed no change in ALP activity compared to cultures in (+/−) medium (Figure 3), but the formation of calcified nodules was significantly inhibited by alizarin red S staining (Figure 4). This may indicate that changes in factors other than ALP are involved in the suppression of osteogenic differentiation and the maintenance of multipotency. Although it has been reported that activation of Wnt/β-catenin signaling induced by Wnt-3a is important for the construction of iPS cells [39], Wnt/β-catenin signaling was not involved in the increase in the ALP activity of HPLFs used in this study. These findings suggest that further investigation of the pathways involved in the maintenance of pluripotency is necessary.

The most abundant matrix protein in bone tissue is collagen, of which Type-I collagen accounts for 95%. Lysyl oxidase accelerates the intermolecular covalent bonding of collagen and contributes to the maintenance of bone strength [40]. The periodontal ligament is lined with Sharpey fibers, which are responsible for anchoring the teeth in place, and it is possible that Type-III collagen characterizes these fibers [41]. The results of alizarin red S staining (Figure 4) showed that culture in (+/−) medium promoted osteogenic differentiation of HPLFs, which required the formation of an adequate matrix. Interestingly, the expression of the *Type-I collagen* gene in HPLFs cultured in (+/-) medium was significantly decreased compared to that in (−/−) medium (Figure 5D). The expression of *Type-I collagen* gene was significantly increased in the culture with (+/+) medium compared with that with (+/−) medium and was recovered to the same level as that in the culture with (−/−) medium. This suggests that matrix formation by collagen other than Type-I collagen is preferentially induced by culture in (+/−) medium. The collagen composition of the human mandible differs from that of other bone tissues, including the fact that it is more flexible than long bones due to looser collagen cross-linking [42] to withstand daily mastication. Interpretation of the role of collagen in osteogenic differentiation of the periodontal ligament is difficult, but the present results provide a clue to this interpretation.

Osteogenic differentiation of mesenchymal stem cells is regulated by a variety of genes, including *Runx2*, *Osterix*, *Osteopontin* and *Osteocalcin* [43,44]. Culture of HPLFs in (+/−) medium significantly increased gene expression of *Runx2*, *Osteopontin* and *Osteocalcin* compared to culture in (−/−) medium (Figure 5A–C). *Runx2* is an essential gene for osteogenesis and mice lacking *Runx2* die immediately after birth [45]. *Osteopontin* is important for bone remodeling as it promotes the proliferation and migration of mesenchymal stem cells [46] and osteoclasts proliferate abnormally in osteopontin-deficient mice [47]. In addition, *Runx2*, *Osteopontin* and *Osteocalcin* are important genes for osteogenesis, as *Osteocalcin*-deficient mice have impaired normal bone formation [48]. In the present study, the expression of osteogenic differentiation-related genes was significantly reduced in (+/+) medium cultures compared to (+/−) medium cultures (Figure 5A–C). In pulp-derived stem cells, the activation of Wnt/β-catenin signaling causes a decrease in ALP activity [49] and alters the gene expression of *Osteocalcin* [50]. It has also been shown that Wnt/β-catenin signaling is important in the osteogenesis of periodontal tissues, such as altering the expression of *Osteopontin* [51] in stem cells derived from the apical papilla, an early tissue in tooth development. The periodontal ligament is the tissue between the alveolar bone and the cementum. In addition to stem cells, the periodontal ligament contains cementoblasts, which constitute the cementum, and progenitor cells of osteoblasts, which form the alveolar bone. *Cementum protein-1* (*CEMP1*) and *Cementum attachment protein* (*CAP*), proteins extracted from cementum, are cementum-related genes [52]. In addition, it has been reported that periodontal ligament-derived cells express cementum-related genes when stimulated by periodontal tissue-derived cells [53,54]. It has been suggested that *CEMP1* may be preferentially expressed in ALP-positive cells among periodontal ligament-derived cells [55]. It has also been reported that *CEMP1* gene expression in periodontal ligament-derived cells is suppressed by the induction of osteogenic differentiation [54] and peaks after 14 days [56]. However, it has further been reported that *CEMP1* and *CAP* gene expression levels do not correlate with *ALP* expression levels in different human individuals from whom periodontal ligament-derived stem cells are extracted [54]. Therefore, the role of cementum-related genes in periodontal ligament cells remains to be elucidated because the expression pattern of cementum-related genes varies depending on the characteristics of the cell population extracted from the periodontal ligament. ALP activity of HPLFs was significantly higher in (−/−) medium than in (+/−) medium (Figure 3), whereas *CAP* gene expression was not significantly different between the four mediums (Figure S3, Table S1). Thus, the *CAP* gene in HPLFs used in this study was independent of ALP activity. The expression of cementum-related genes, such as *CAP* and *CEMP1*, has not yet been established, as cementum-related genes have been reported to be expressed in Hertwig’s epithelial root sheath cells involved in enamel formation [52], and their evaluation should be treated with caution.

The periodontal ligament is a soft tissue composed of collagen and strongly expresses *Postn* [57]. Because periostin is not expressed in periodontal bone tissues such as dentin and cementum, it is a necessary protein for the maintenance of the periodontal ligament [58]. The expression of periostin increases under mechanical stresses such as mastication [59], suggesting that it is also important for periodontal ligament remodeling. This study shows that the expression of Type-I collagen and *Postn* genes was significantly decreased in HPLFs cultured in (+/−) medium compared to (−/−) medium, and the expression levels of these genes were significantly increased in (+/+) medium compared to (+/−) medium (Figure 5D,E). Thus, HPLFs increased the expression of *Type-I collagen* and *Postn* genes in association with the depression of osteogenic differentiation by the addition of Wnt-3a.

The expression of *Mkx* and *Scx* genes is highly expressed in tendon cells and is important in the maintenance of tendons [60,61]. Because the periodontal ligament is responsible for anchoring the tooth in its normal position by connecting the alveolar bone and cementum with Sharpey fibers, and its role is similar to that of tendons, the gene expression levels of *Mkx* and *Scx* were investigated. The *Mkx* affects the maintenance of adequate periodontal ligament width and cell morphology in the periodontal ligament [10]. It is also closely involved in the maintenance of periodontal tissue homeostasis, as alveolar bone is resorbed by abnormal activation of osteoclasts in *Mkx* knockout mice. The present study suggests that the expression of the *Mkx* gene may not be involved in the osteogenic differentiation of HPLFs or in the suppression of osteogenic differentiation (Figure 6A). The *Scx* gene has been reported to be involved in the inhibition of osteogenesis in the periodontal ligament [11,62] and is thought to be important in maintaining the width of the periodontal ligament. In the present study, we found that the expression of the *Scx* gene was increased when HPLFs were cultured in (+/+) medium compared to other media (Figure 6B). It has also been reported that overexpression of the *Scx* gene suppresses osteogenic differentiation in periodontal ligament-derived cells by inhibiting the expression of *Postn*, *Osteopontin* and *Osteocalcin* [11]. Therefore, the inhibition of osteogenic differentiation in HPLF cultures in (+/+) medium could be the result of the suppression of the expression of osteogenic differentiation-related genes due to the high expression of *Scx* induced by Wnt-3a.

## 5. Conclusions

It can be concluded from this study that the activation of Wnt/β-catenin signaling induced by Wnt-3a in HPLFs is an important signal responsible for the maintenance of periodontal ligament homeostasis by inhibiting osteogenic differentiation through the upregulation of the expression of *Scx* and *Postn* genes. This is the first report to investigate the relationship of the tendon-specific gene *Scx* via Wnt/β-catenin signaling in HPLFs.

## Figures and Tables

**Table 1:** Classification of culture medium.

**Abbreviation**	−/− (DMEM only)	−/+	+/−	+/+
**Osteogenic factors**	−	−	+	+
**Wnt−3a (50 ng/mL)**	−	+	−	+

**Table 2:** The primers used in this study.

GENE	ID	Sequence	
*18S rRNA*	NR_145820.1	Forward	GTAACCCGTTGAACCCCATTC
		Reverse	CCATCCAATCGGTAGTAGCG
*Runx2*	NM_001015051.3	Forward	TTCGTCAGGATCCTATCAGTTTC
		Reverse	TTTAATAGCGTGCTGCCATTC
*Osteopontin*	NM_000582.2	Forward	CTGGATGACCAGAGTGCTGA
		Reverse	TTGCTCTCATCATTGGCTTTC
*Osteocalcin*	NM_199173.6	Forward	CCTCACACTCCTCGCCCTATT
		Reverse	CCCTCCTGCTTGGACACAAA
*Type-I collagen*	NM_000088.4	Forward	GGGATTCCCTGGACCTAAAG
		Reverse	TCCCTGAGCTCCAGCCTCTCC
*Periostin*	NM_001135934.2	Forward	AAGCTCAGGATCCTATCAGTTTC
		Reverse	TGGTTGGCACAAATAATGTCC
*Mkx*	NM_001242702.2	Forward	TTACAAGCACCGTGACAACC
		Reverse	AAGCCGACGTCTTGCATTAG
*Scx*	NM_001080514.3	Forward	GAGAACACCCAGCCCAAAC
		Reverse	CTGCGAATCGCTGTCTTTCT

## Supplementary Materials

The following supporting information can be downloaded at: https://www.mdpi.com/article/10.3390/biomimetics7040224/s1, Figure S1: Fluorescence observation of the nuclear translocation of β-catenin upon stimulation with Wnt-3a. HPLFs were seeded at a density of 1.25 × 10^4^ cells/cm^2^ and the medium was changed after one day. Fifteen min later, cells were observed. The cells were fixed and stained with Alexa Flour^®^ 488-labeled phalloidin for actin (green), anti-β-catenin for β-catenin (red) and DAPI for cell nuclei (blue). Scale bars indicate 200 μm. Figure S2: Results of ALP activity measurement when HPLFs were cultured for 21 days. HPLF was cultured in (−/−) and (+/−) medium and ALP activity was assayed. On the 14th day of culture, ALP activity was significantly increased in the (−/−) medium. In (+/−) medium, no change in ALP activity was observed from the 5th day of incubation, whereas in (−/−) medium, ALP activity continued to increase as a function of time. Error bars; mean ± S.D, ** *p* < 0.01, (n ≧ 3).; Figure S3: Gene expression analysis by Real Time Quantitative PCR (Cementum related genes). The expression of CAP (Cementum attachment protein) gene was determined after 14 days of incubation of HPLF in each medium. There was no significant difference between the mediums. Data represent the means ± S.D; Table S1: The primers of genes.
