# Supplementary material for: Wnt/β-Catenin Signaling Inhibits Osteogenic Differentiation in Human Periodontal Ligament Fibroblasts
Source: Biomimetics (Basel). 2022 Dec 3;7(4):224. doi: 10.3390/biomimetics7040224 (PMC9776043; doi:10.3390/biomimetics7040224)
Supplement: Supplementary file 1 [file biomimetics-07-00224-s001.zip › biomimetics-2037033-supplementary.pptx]

## Slide 1
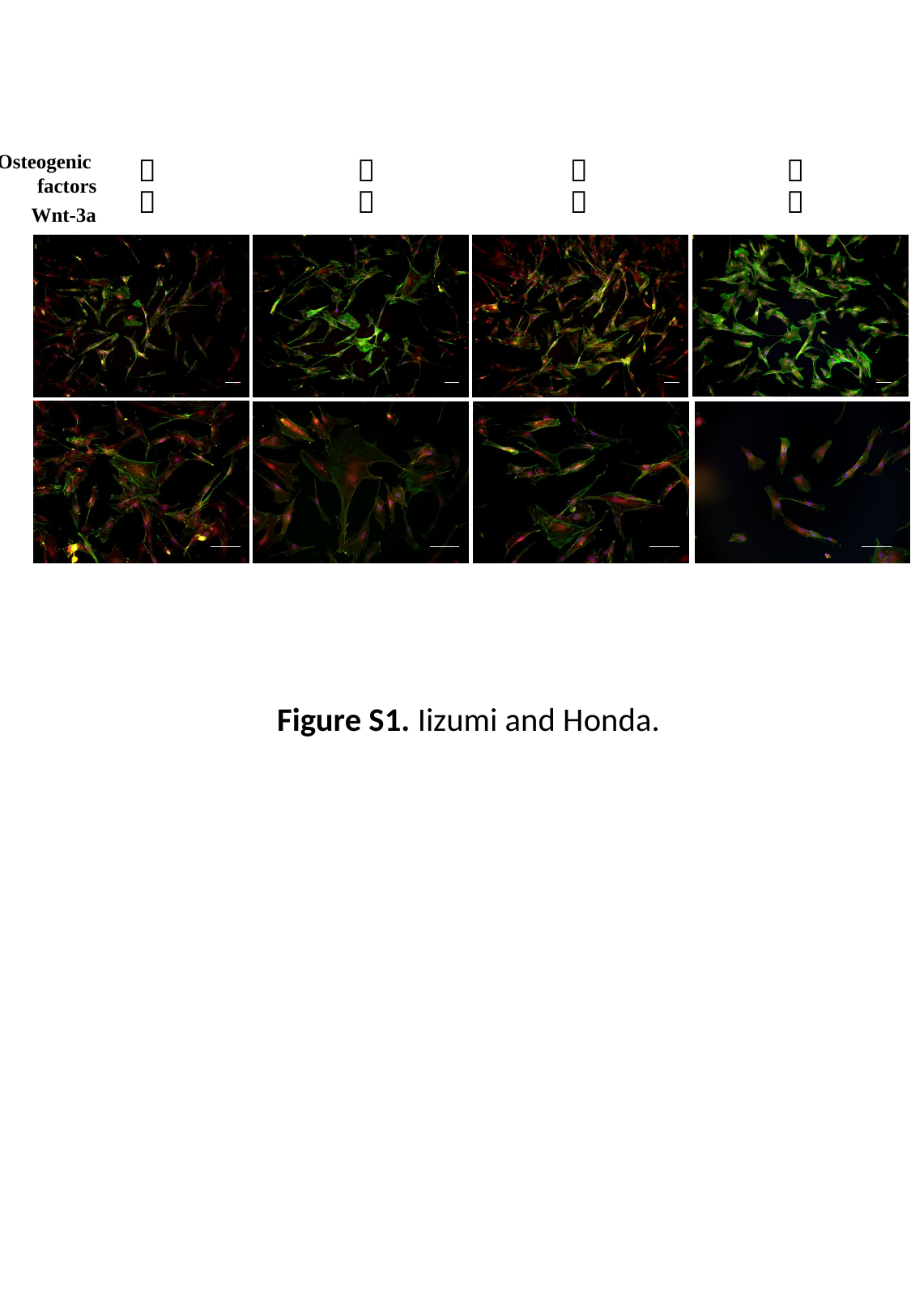

Osteogenic
factors
－
＋
－
＋
－
－
＋
＋
Wnt-3a
Figure S1. Iizumi and Honda.

## Slide 2
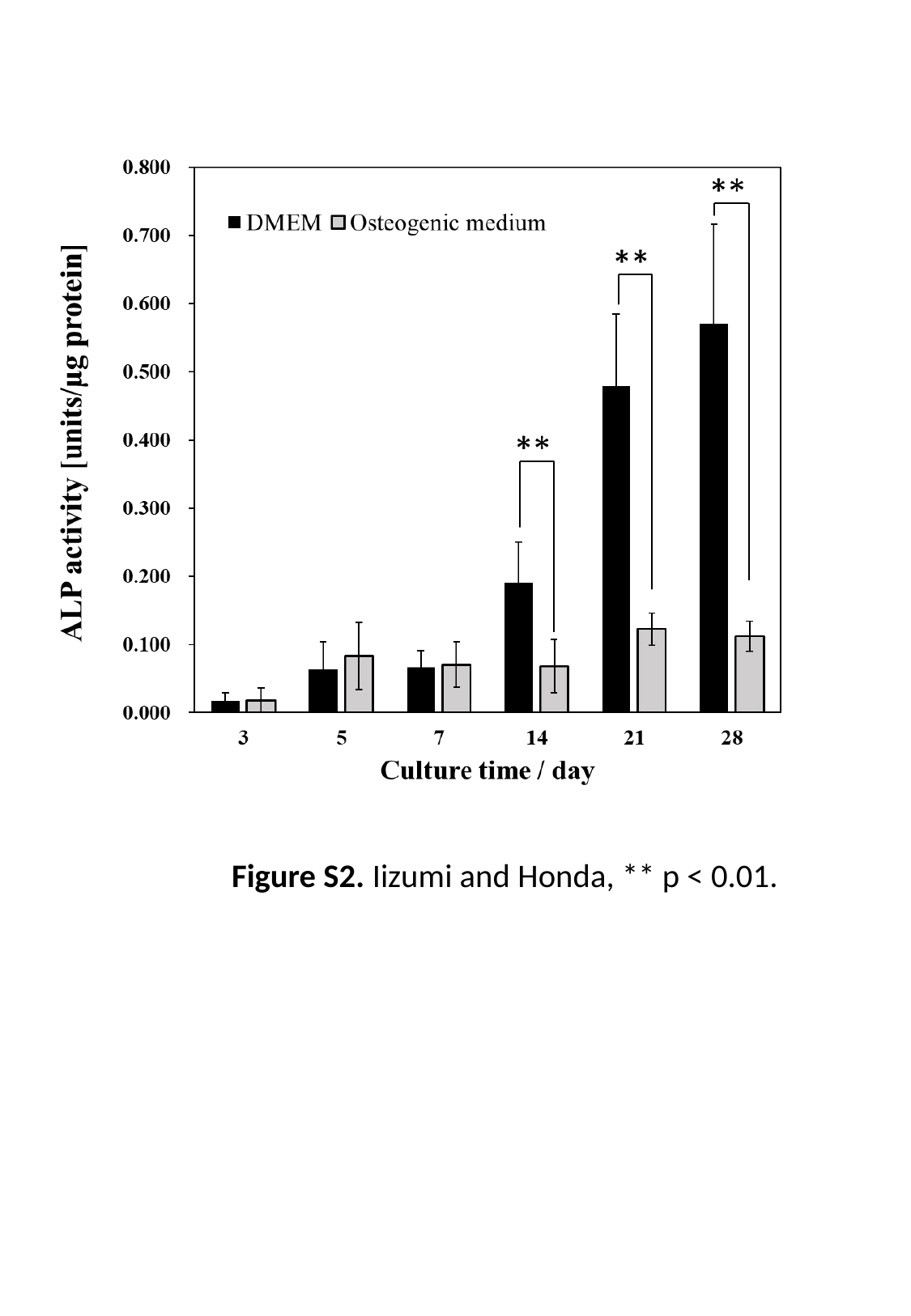

Figure S2. Iizumi and Honda, ** p < 0.01.

## Slide 3
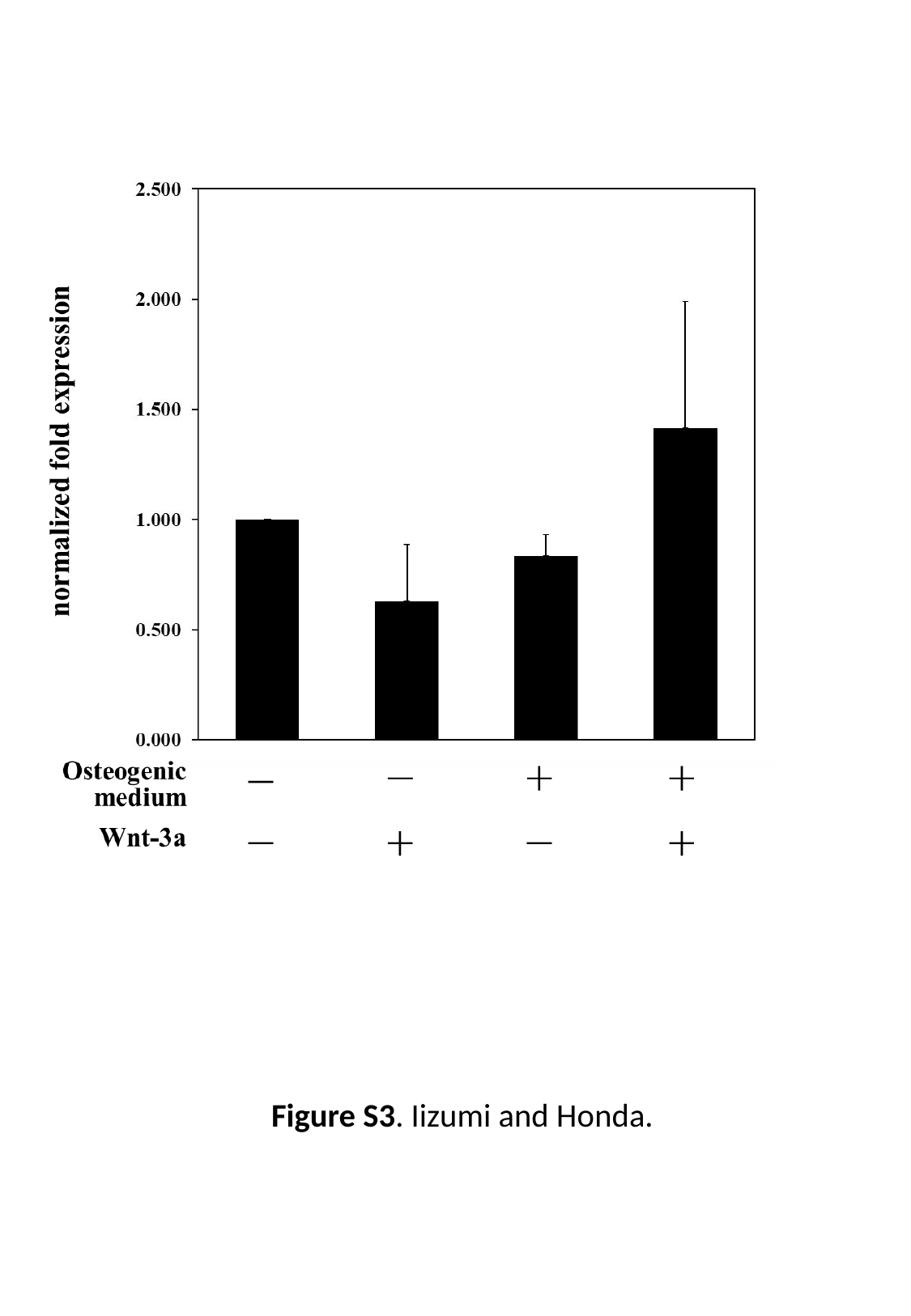

Figure S3. Iizumi and Honda.
